# Endogenous estradiol contributes to vascular endothelial dysfunction in premenopausal women with type 1 diabetes

**DOI:** 10.1186/s12933-023-01966-6

**Published:** 2023-09-07

**Authors:** Abigayle B. Simon, Cassandra C. Derella, Marsha Blackburn, Jeffrey Thomas, Lawrence C. Layman, Matthew S. Nicholson, Jennifer Waller, Ahmed Elmarakby, Karim M. Saad, Ryan A. Harris

**Affiliations:** 1 Georgia Prevention Institute, Medical College of Georgia, Augusta University, 1120 15th Street, HS-1707, Augusta, GA 30912 Georgia; 2 Section of Reproductive Endocrinology, Infertility, & Genetics, Department of Obstetrics & Gynecology, Medical College of Georgia, Augusta University, Augusta, GA Georgia; 3 Department of Endocrinology, Medical College of Georgia, Augusta University, Augusta, GA Georgia; 4 Department of Biostatistics and Data Science, Medical College of Georgia, Augusta University, Augusta, GA Georgia; 5 Department of Oral Biology and Diagnostic Sciences, Dental College of Georgia, Augusta University, Augusta, GA Georgia

**Keywords:** Diabetes, Endothelial function, Estrogen, Oral contraceptives

## Abstract

**Background:**

Endogenous estrogen is cardio-protective in healthy premenopausal women. Despite this favorable action of estrogen, animal models depict a detrimental effect of estradiol on vascular function in the presence of diabetes. The present study sought to determine the role of endogenous estradiol on endothelial function in women with type 1 diabetes.

**Method:**

32 women with type 1 diabetes (HbA_1c_ = 8.6 ± 1.7%) and 25 apparently healthy women (HbA_1c_ = 5.2 ± 0.3%) participated. Flow-mediated dilation (FMD), a bioassay of nitric-oxide bioavailability and endothelial function was performed during menses (M) and the late follicular (LF) phase of the menstrual cycle to represent low and high concentrations of estrogen, respectively. In addition, a venous blood sample was collected at each visit to determine circulating concentrations of estradiol, thiobarbituric acid reactive substances (TBARS), and nitrate/nitrite (NOx), biomarkers of oxidative stress and nitric oxide, respectively. Data were collected in (1) 9 additional women with type 1 diabetes using oral hormonal birth control (HBC) (HbA_1c_ = 8.3 ± 2.1%) during the placebo pill week and second active pill week, and (2) a subgroup of 9 demographically matched women with type 1 diabetes not using HBC (HbA_1c_ = 8.9 ± 2.1%).

**Results:**

Overall, estradiol was significantly increased during the LF phase compared to M in both type 1 diabetes (Δestradiol = 75 ± 86 pg/mL) and controls (Δestradiol = 71 ± 76 pg/mL); however, an increase in TBARS was only observed in patients with type 1 diabetes (ΔTBARS = 3 ± 13 µM) compared to controls (ΔTBARS = 0 ± 4 µM). FMD was similar (*p* = 0.406) between groups at M. In addition, FMD increased significantly from M to the LF phase in controls (*p* = 0.024), whereas a decrease was observed in type 1 diabetes. FMD was greater (*p* = 0.015) in patients using HBC compared to those not on HBC, independent of menstrual cycle phase.

**Conclusion:**

Endogenous estradiol increases oxidative stress and contributes to endothelial dysfunction in women with diabetes. Additionally, HBC use appears to be beneficial to endothelial function in type 1 diabetes.

## Introduction

Cardiovascular disease (CVD) remains the leading cause of death throughout the world [1], and there are various health disparities related to the prevalence of CVD. Specifically, the rate of developing CVD is lower in premenopausal women compared to age-matched men [2]. Moreover, type 1 diabetes increases the prevalence of CVD [3]. In fact, individuals with type 1 diabetes can have up to an 8 times greater risk of CVD compared to their age-matched healthy counterparts [4].

In general, apparently healthy premenopausal women are protected against CVD compared with men and postmenopausal women, a benefit that is likely due to the presence of circulating estrogens [5]. Interestingly, this cardio-protective benefit of female sex appears to be lost in the presence of diabetes. In fact, women with type 1 diabetes are 2–3 times more likely to develop CVD compared to their male counterparts [6]. Additionally, women with type 1 diabetes have a 40% higher rate of all-cause mortality compared to men with type 1 diabetes [7], albeit exhibiting a more favorable cardio-metabolic risk factor profile [8].

During a regular menstrual cycle, circulating concentrations of estradiol are low during menses and peak in the late follicular phase just prior to ovulation [9]. Activation of estrogen receptors (α and β) and GPR30 receptors within the endothelium not only facilitate the endothelial release of nitric oxide (NO), downstream signaling pathways via genomic and non-genomic effects [10] contribute to further smooth muscle relaxation and promote additional vasodilatory properties [11]. In addition, estrogens have antioxidant properties [12] that reduce oxidative stress and contribute to increases in NO bioavailability. The flow-mediated dilation (FMD) test is recognized as a bioassay of NO bioavailability and can predict future CVD and cardiovascular events [13]. Accordingly, although the increase in FMD from menses to the late follicular phase of the menstrual cycle has been observed in healthy women [14–18] and has been attributed to the increases in estradiol [11], others have found no difference in FMD across the menstrual cycle [19]. In addition, data from animal models of type 1 diabetes have demonstrated a vasoconstrictive response to estradiol that is mediated by an increase in oxidative stress [20]. Despite the epidemiological and experimental data, the role of estradiol on vascular endothelial function in women with type 1 diabetes has yet to be investigated.

In the United States, 88% of women ages 15 to 44 years have used at least one form of hormonal birth control (HBC) throughout their lifetime [21]; approximately 22% of whom specifically use oral contraceptives as their preferred method of HBC [22]. HBC works by delivering a low dose of exogenous estrogen and/or progesterone [23], which reduces follicle-stimulating hormone (FSH) and luteinizing hormone (LH), and prevents the increase in endogenous estradiol and subsequent ovulation [23]. Although there is scant evidence that healthy, premenopausal women using HBC may be at a greater risk for developing cardiovascular complications compared to women not using HBC [24], the role of suppressing circulating concentrations of estradiol using HBC on vascular endothelial function in women with type 1 diabetes has yet to be elucidated.

Thus, the present study sought to test the hypotheses that (1) the increase in estradiol from menses to the late follicular phase of the menstrual cycle would impair endothelial function in women with type 1 diabetes compared to an improvement in endothelial function observed in healthy controls, and (2) the use of HBC would prevent the increase in endogenous estradiol and contribute to a higher endothelial function in women with type 1 diabetes compared to women with type 1 diabetes who do not use HBC.

## Methods

### Experimental design

All participants reported to the Laboratory of Integrative Vascular and Exercise Physiology (LIVEP) in the Georgia Prevention Institute at Augusta University for a preliminary screening visit that consisted of the informed consent process, body composition assessments, blood pressure, and anthropometric measures. A single stick blood draw was performed to obtain C-reactive protein (CRP), glycated hemoglobin (HbA_1c_), and a lipid panel using standard core laboratory techniques (Laboratory Corporation of America Holdings, Birmingham, AL). Height and mass were determined using a stadiometer and standard platform scale (CN20, DETECTO, Webb City, MO), respectively, and were used to calculate body mass index (BMI). Total body fat was determined using dual energy X-ray absorptiometry (QDR-4500 W; Hologic, Waltham, MA).

Following the preliminary visit, all participants returned for two experimental visits: during the menses and late follicular phase of the menstrual cycle, which coincided with low and high concentrations of endogenous estradiol, respectively. At the cessation of the first experimental visit (menses), participants were given an ovulation predictor kit (OPK, Clearblue, Procter & Gamble, Cincinnati, OH) to help predict the luteinizing hormone (LH) surge and scheduling of their second experimental visit (late follicular phase). Based on the manufacturer’s recommendations, participants informed the study staff when they received a flashing smiley face from the OPK and were scheduled for testing at the LIVEP the following day. Patients with type 1 diabetes (n = 9) and controls (n = 5) who did not receive a smiley face were tested during days 13–16 of their menstrual cycle to try to capture the physiological increase in circulating concentrations of estradiol.

For each experimental visit, participants arrived at the LIVEP in the morning following an overnight fast and having abstained from caffeine, smoking or any tobacco use, or moderate-to-vigorous exercise for at least 12 h prior to testing. Patients with type 1 diabetes were instructed to maintain their basal insulin regimen during each visit to avoid variations between visits. On each experimental day, a venous blood sample was collected to assess circulating concentrations of insulin and various sex hormones including estradiol, LH, and progesterone (Laboratory Corporation of America Holdings, Birmingham, AL). Biomarkers of oxidative stress and NO were also assessed, and blood glucose was determined using a point-of-care glucometer (Accu-Chek, Indianapolis, IN).

Patients that were currently using HBC were recruited to test the effects of suppressing the endogenous production of estradiol. For the HBC (+) group, the first experimental visit was scheduled during the placebo pill week, and the second experimental visit was scheduled during the second active pill week. This length of time between experimental visits was consistent with the time between visits in the HBC (-) group. For the subgroup analysis, the first experimental visit (menses/placebo pill week) will be represented as M/P and the second experimental visit (late follicular/active pill week) will be represented as LF/A.

### Participant characteristics

Thirty-two premenopausal women with a clinical diagnosis of type 1 diabetes were recruited from the Department of Endocrinology at Augusta University or from the community via word of mouth. In addition, 25 apparently healthy, premenopausal women that were recruited from the community participated in this study. All participants were required to have a normal menstrual cycle interval of 25–35 days for at least three previous cycles, to not only assist with the timing of the second visit, but also to exclude women with irregular menstrual cycles. In addition, to provide proof-of-concept that suppressing endogenous production can be vascular protective, a subgroup of 9 additional women with type 1 diabetes who were currently using oral forms of HBC (HBC (+)) were recruited and demographically matched to 9 women with type 1 diabetes not using HBC (HBC (-)) from the main study. Participants were excluded if they reported a history of hepatic, renal, or overt CVD, uncontrolled hypertension (i.e., systolic/diastolic > 140/90 mm Hg), polycystic ovary syndrome, oligomenorrhea based on self-report, or proteinuria. Participants using any medications that interacted with estrogen metabolism (other than HBC) were excluded. In addition, women who were pregnant or those trying to become pregnant were excluded from the study. Women experiencing any vascular-related complications of diabetes or those with an HbA_1c_ of > 12% were also excluded. All study protocols were approved by the Institutional Review Board at Augusta University and this study was registered on clinicaltrials.gov (NCT03436992).

### Vascular endothelial function

During both experimental visits, the primary outcome was endothelial-dependent vasodilation and was determined using the brachial artery flow-mediated dilation (FMD) test in accordance with the most recent guidelines [25]. Briefly, participants laid in a rested, supine position for at least 15 min to ensure stable blood flow and a hemodynamic steady state. Using a 12 MHz linear transducer, simultaneous B-mode and blood velocity profiles (duplex mode) of the brachial artery were obtained (Logiq 7, GE Medical Systems, Milwaukee, WI). After 30 s of baseline data collection, the forearm occlusion cuff (E-20 rapid cuff inflator; D.E. Hokanson) that was placed immediately distal to the medial epicondyle was rapidly inflated to 250 mm Hg. Following 5 min of forearm occlusion, the cuff was released, and brachial artery diameter and blood velocity were continuously recorded for 2 min. R-wave gating (Accusync 72, Accusync Medical Research Corporation, Milford, CN) was utilized to capture end-diastolic arterial diameters for automated offline analysis of brachial artery vasodilation (Medical Imaging Applications, Coralville, Iowa). FMD (%) is reported as the percent of maximal brachial artery dilation diameter from baseline diameter. Cumulative shear rate (s^− 1^, area under the curve, AUC) was determined using the trapezoidal rule, every 4 s for the first 20 s following cuff release, and every 5 s thereafter for the remainder of the 2-min data collection period. Following completion of the FMD test, baseline measurements were taken for 30 s before 0.4 mg of sublingual nitroglycerin (NTG) (Perrigo, Allegan, MI) was administered. Endothelial-independent vasodilation of the brachial artery was continuously recorded for 8–10 min to ensure the peak response was obtained, and the percent of maximal brachial artery dilation from baseline was used to represent NTG (%). All measurements were made by a reviewer that was blinded to the group/menstrual cycle phase.

### Blood processing and biomarkers of oxidative stress

Following an overnight fast, venous blood samples (~ 30 mL) were collected into EDTA Vacutainer™ systems (BD, Franklin Lakes, NJ) at each visit. All blood samples were centrifuged at 3000 rpm at 4 °C for 10 min to separate plasma. Plasma samples were stored at -80 °C for future analysis. Thiobarbituric acid reactive substances (TBARS) and nitrate/nitrite (NOx) were assessed in plasma as secondary endpoints to determine biomarkers of lipid peroxidation and circulating concentrations of NO, respectively. The Malondialdehyde-Thiobarbituric Acid (MDA-TBA) adduct formed by the reaction of MDA and TBA under high temperature (90–100 °C) and acidic conditions was measured colorimetrically between 530 and 540 nm according to the manufacturer’s specifications (Cayman Chemicals, Ann Arbor, MI). In addition, total NOx concentration was determined using a two-step process according to the manufacturer’s specifications (Cayman Chemicals, Ann Arbor, MI). The detection range for TBARS and NOx was between 0.0625 and 50 µM and 5–35 µM, respectively.

### Statistical analysis

Statistical analyses were performed using SAS 9.4. All data are expressed as mean ± standard error of mean (SEM) unless otherwise noted. Descriptive statistics for demographic and baseline clinical variables were determined during menses and the follicular phase of the menstrual cycle. For demographic and baseline clinical variables, chi-square tests or two-sample t-tests were used to examine differences. To examine differences in estradiol, FMD, NTG, TBARS, and NOx during menses and the late follicular phase of the menstrual cycle, mixed models were used to determine changes over time and differences between groups. Fixed effects in each model included group status (type 1 diabetes vs. control) and menstrual cycle phase (menses vs. late follicular phase) and the two-factor interaction between group and menstrual cycle phase. Post hoc pairwise comparisons using the two-factor interaction of group and menstrual cycle phase were performed within group, between menstrual cycle phase, and within menstrual cycle phase between groups using a Bonferroni adjustment to the overall alpha level for the number of comparisons. A similar set of analyses were performed for estradiol, FMD, and NTG on a subset of women with type 1 diabetes who were using HBC (HBC (+)) or not using HBC (HBC (-)). Effect sizes for differences in FMD and TBARS are reported as Cohen’s *d* values to represent small (Cohen’s *d* = 0.2), medium (Cohen’s *d* = 0.5), and large (Cohen’s *d* = 0.8) effect sizes [26]. Statistical significance was set at *p* < 0.05.

## Results

Participant demographics and clinical laboratory values for the main analysis are presented in Table 1. The average age of diabetes diagnosis was 12 ± 6 years (range 3–27 years). A similar (*p* = 0.213) proportion of Black participants were enrolled in each group. As expected, HbA_1c_ and fasting blood glucose was higher (*p* < 0.001) in patients compared to controls. Importantly, fasting blood glucose (*p* = 0.261) and circulating concentrations of insulin (*p* = 0.619) were similar throughout the menstrual cycle in patients with diabetes. Additionally, women with type 1 diabetes exhibited significantly higher (*p* = 0.008) CRP compared with healthy controls. No significant differences in any other participant demographics or laboratory values were observed between groups.

**Table 1:** Participant Characteristics and Clinical Laboratory Values

Variable	Type 1 Diabetes	Controls	p-value
	(n = 32)	(n = 25)	
**Demographics**			
Age (y)	25 ± 6	24 ± 6	0.365
Time Since Diagnosis (y)	13 ± 7		
Height (cm)	163.5 ± 7.0	165.4 ± 5.8	0.273
Weight (kg)	72.8 ± 17.6	68.4 ± 15.8	0.336
BMI (kg/m^2^)	27.2 ± 5.7	24.5 ± 4.7	0.060
MAP (mm Hg)	87 ± 8	83 ± 7	0.069
**Clinical Laboratory Values**			
CRP (mg/L)	5.4 ± 8.3	1.2 ± 1.3	**0.008**
TC (mg/dL)	178 ± 41	163 ± 35	0.161
HDL (mg/dL)	58 ± 17	53 ± 13	0.228
LDL (mg/dL)	98 ± 42	88 ± 39	0.337
TRIG (mg/dL)	76 ± 45	63 ± 28	0.158
TC/HDL ratio	3.2 ± 1.1	3.2 ± 0.8	0.788
HbA1c (%)	8.6 ± 1.7	5.2 ± 0.3	**< 0.001**
FBG (mg/dL) – Menses	153 ± 79	85 ± 9	**< 0.001**
FBG (mg/dL) – Late Follicular	168 ± 95	85 ± 10	**< 0.001**
Insulin (uIU/mL) – Menses	3.1 ± 9.8	8.1 ± 4.6	**0.015**
Insulin (uIU/mL) – Late Follicular	2.9 ± 7.8	9.4 ± 5.8	**< 0.001**
Estradiol (pg/mL) – Menses	40 ± 25 (9-114)	30 ± 15 (8–72)	0.084
Estradiol (pg/mL) – Late Follicular	115 ± 82* (25–356)	100 ± 84* (17–391)	0.521
LH (mIU/mL) – Menses	6 ± 4 (1–21)	7 ± 2 (3–14)	0.783
LH (mIU/mL) – Late Follicular	12 ± 7* (3–36)	16 ± 13* (7–56)	0.099
Progesterone (ng/mL) – Menses	0.4 ± 0.4 (0.1–1.5)	0.3 ± 0.2 (0.1–0.8)	0.469
Progesterone (ng/mL) – Late Follicular	0.9 ± 1.5 (0.1-7.0)	1.0 ± 1.6* (0.1–6.3)	0.799

Data are presented as mean ± SD (Range); independent samples t test. Bold values indicate statistically significant differences between groups. *Significant difference from Menses within group. BMI = Body Mass Index, MAP = Mean Arterial Pressure, CRP = C-Reactive Protein, TC = Total Cholesterol, HDL = High-Density Lipoprotein, LDL = Low-Density Lipoprotein, TRIG = Triglycerides, HbA1c = Hemoglobin A1c, FBG = Fasting Blood Glucose, LH = Luteinizing Hormone

### Estradiol and endothelial function during the menstrual cycle in type 1 diabetes and controls

**Fig. 1:**
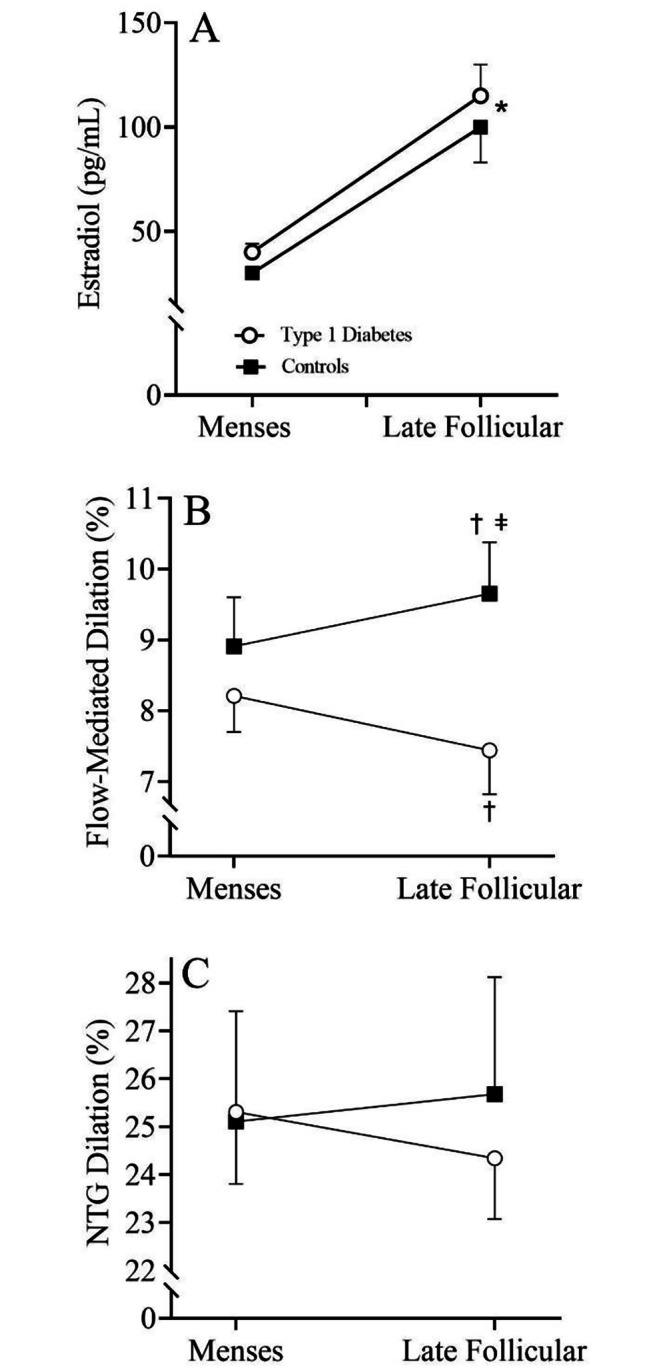
**(A)** Circulating concentrations of estradiol across the menstrual cycle in women with type 1 diabetes (open circle) and healthy controls (closed circle); n = 57 (Type 1 Diabetes = 32, Controls = 25). Vascular endothelial function across the menstrual cycle in women with type 1 diabetes and healthy controls using **(B)** Flow-mediated dilation; n = 57 (Type 1 Diabetes = 32, Controls = 25) and **(C)** Nitroglycerin dilation; n = 34 (Type 1 Diabetes = 19, Controls = 15). * indicates a significant time main effect. † indicates a significant difference from menses. ǂ indicates a significant difference from type 1 diabetes during the late follicular phase. Repeated measures ANOVA

Testing during the M and LF phases occurred during days 4 ± 1 and 14 ± 2, respectively for healthy controls and 4 ± 1 and 14 ± 2, respectively for individuals with type 1 diabetes. Figure 1A illustrates the circulating concentration of estradiol across the menstrual cycle in both type 1 diabetes and healthy controls. No differences in the estradiol response between groups was observed; however, there was a significant increase in estradiol (*p* < 0.001) during the LF phase in both groups. In addition, a significant time main effect (*p* < 0.001) for both LH and progesterone was observed. LH and progesterone increased significantly (*p* < 0.007) from menses to the late follicular phase of the menstrual cycle in both groups. No differences (all *p* > 0.05) in LH or progesterone were observed between groups (Table 1). Figure 1B illustrates the FMD response during the menstrual cycle in women with type 1 diabetes and healthy controls. A significant group by menstrual cycle interaction (*p* = 0.042) was observed. Specifically, FMD was similar between groups at menses (*p* = 0.406); however, healthy controls exhibited a significantly higher (*p* = 0.024; Cohen’s *d* = 3.56) FMD during the late follicular phase when compared to a decrease in FMD throughout the menstrual cycle in type 1 diabetes. The parameters of the FMD test in patients with type 1 diabetes compared to controls are presented in Table 2. There were no differences (all *p* > 0.05) in baseline diameter, peak diameter, and shear rate between groups. Similar to the FMD response, a significant group by menstrual cycle interaction (*p* = 0.038) was observed for FMD/Shear. Specifically, a significant increase in FMD/Shear from menses to the late follicular phase was observed in controls, whereas a decrease was observed in type 1 diabetes during the late follicular phase. Figure 1C illustrates the vasodilatory response to NTG across the menstrual cycle in women with type 1 diabetes and healthy controls. No differences (*p* = 0.557) in endothelial-independent vasodilation were observed between groups or throughout the menstrual cycle.

**Table 2:** Parameters of the FMD test throughout the menstrual cycle in patients with Type 1 Diabetes and Controls

Variable	Menses			Late Follicular		
	Type 1 Diabetes	Controls	p-value	Type 1 Diabetes	Controls	p-value
Baseline diameter (cm)	0.300 ± 0.033	0.299 ± 0.029	0.831	0.301 ± 0.035	0.297 ± 0.033	0.690
Peak diameter (cm)	0.325 ± 0.035	0.325 ± 0.031	0.991	0.322 ± 0.033	0.326 ± 0.035	0.725
Shear (s^− 1^ AUC)	49,321 ± 20,062	46,903 ± 15,194	0.619	48,694 ± 16,337	47,949 ± 15,270	0.861
FMD/Shear (%/s^− 1^ AUC)	0.182 ± 0.068	0.206 ± 0.100	0.284	0.155 ± 0.059	0.219 ± 0.110	**0.007**

Values are mean ± SD. AUC = Area under the curve, FMD = Flow-mediated dilation. Bold values indicate between group significance at each menstrual cycle phase

### Circulating biomarkers of oxidative stress and NO concentration during the menstrual cycle in type 1 diabetes and controls

**Fig. 2:**
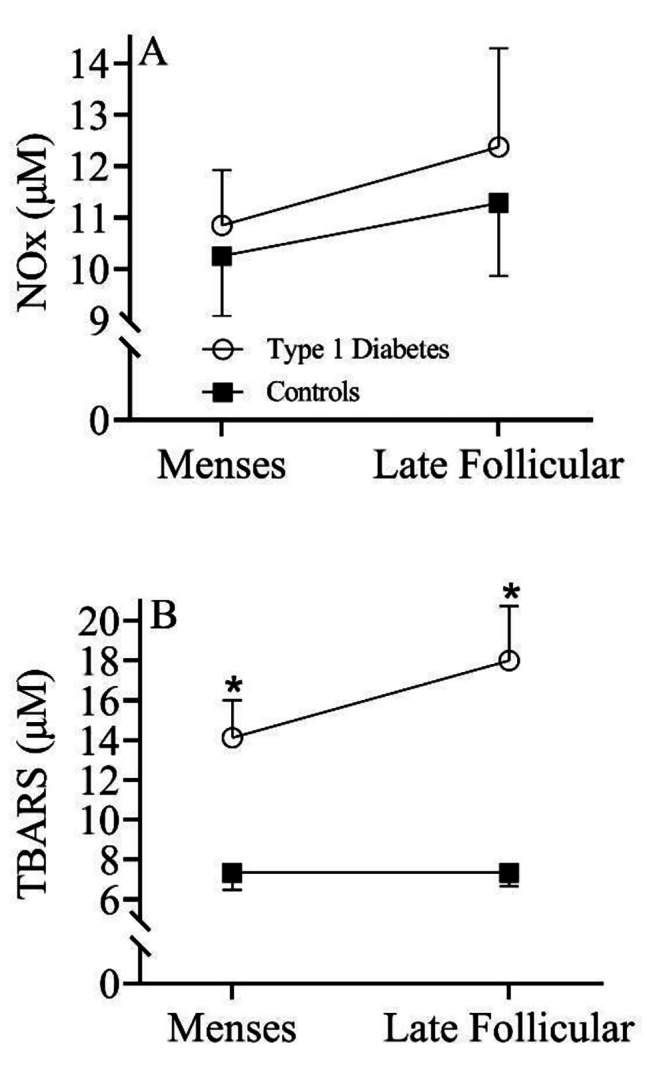
Circulating concentrations of **(A)** NOx; n = 54 (Type 1 Diabetes = 29, Controls = 25) and **(B)** TBARS; n = 54 (Type 1 Diabetes = 29, Controls = 25) across the menstrual cycle in women with type 1 diabetes (open circle) and healthy controls (closed circle). Repeated measures ANOVA. * indicates a significant difference between groups

Figure 2A illustrates the NOx response as a measure of circulating NO throughout the menstrual cycle in both groups. A similar (*p* > 0.05) increase in NOx was observed from M to the LF phase of the menstrual cycle in both women with type 1 diabetes and controls. Figure 2B illustrates the TBARS response as a measure of oxidative stress throughout the menstrual cycle in both groups. During both menses (*p* = 0.002; Cohen’s *d =* 8.13) and the late follicular phase (*p* < 0.001; Cohen’s *d =* 11.05) of the menstrual cycle, TBARS was significantly greater in women with type 1 diabetes compared to controls. In addition, the change in TBARS from menses to the late follicular phase in women with type 1 diabetes was higher (*p* = 0.133) (ΔTBARS = 3 ± 13µM) compared to controls (ΔTBARS = 0 ± 4µM).

### Effects of HBC on estradiol and endothelial function in women with type 1 diabetes

**Fig. 3:**
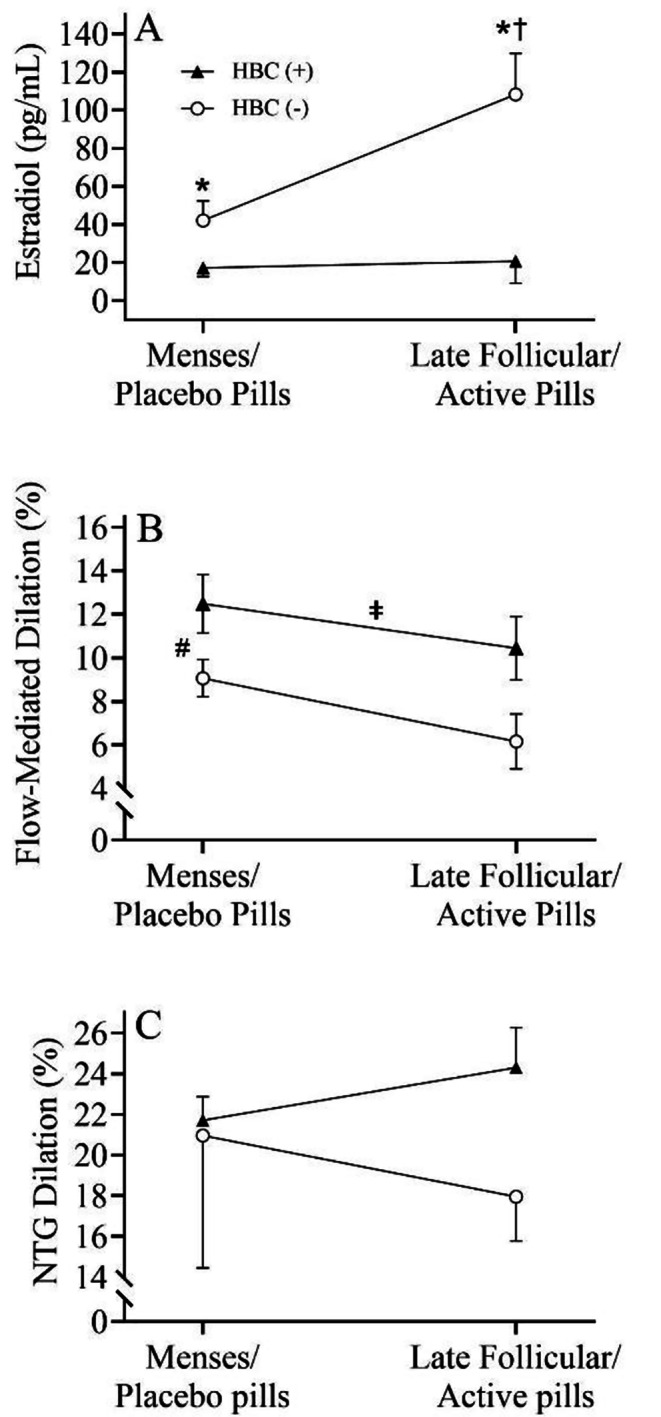
**(A)** Circulating concentrations of estradiol across the menstrual cycle in women with type 1 diabetes using HBC ((HBC (+)) (closed triangle) and women with type 1 diabetes not using HBC ((HBC (-)) (open circle). * indicates significance from HBC (+) at each phase. † indicates significance from HBC (-) at menses. Repeated measures ANOVA; n = 18 (HBC (+) = 9, HBC (-) = 9). Vascular endothelial function across the menstrual cycle in women with type 1 diabetes using HBC ((HBC (+)) and women with type 1 diabetes not using HBC ((HBC (-)) using **(B)** Flow-mediated dilation; n = 18 (HBC (+) = 9, HBC (-) = 9) and **(C)** Nitroglycerin dilation; n = 9 (HBC (+) = 6, HBC (-) = 3). ǂ indicates a significant group main effect. # indicates a significant menstrual cycle phase main effect. Repeated measures ANOVA

Patient demographics and clinical laboratory values for the proof-of-concept subgroup analyses are presented in Table 3. No differences (all *p* > 0.05) in participant demographics were observed between groups. Importantly, fasting blood glucose was similar across visits in both HBC (+) (*p* = 0.502) and HBC (-) (*p* = 0.337). In addition, circulating concentrations of insulin were similar across visits in HBC (+) (*p* = 0.923) and HBC (-) (*p* = 0.302). Testing in the HBC (-) group during M/P and LF/A occurred on days 3 ± 1 and 16 ± 2, respectively, and on placebo pill day 4 ± 2 and active pill day 11 ± 1 for the HBC (+) group. Figure 3A illustrates the significant group by menstrual cycle interaction (*p* = 0.015) for circulating estradiol across the menstrual cycle in both HBC (+) and HBC (-). Specifically, concentrations of estradiol were higher in the HBC (-) group compared to HBC (+) during M/P (*p* = 0.008) and LF/A (*p* = 0.002). In addition, there was a significant (*p* = 0.001) increase in estradiol from M/P to LF/A in the HBC (-) group, whereas no change (*p* = 0.839) was observed in the HBC (+) group. Further, a significant (*p* = 0.047) group x menstrual cycle interaction was observed for LH. Specifically, LH was similar (*p* = 0.203) at M/P between groups; however, the HBC (-) group exhibited a significantly (*p* = 0.017) greater increase in concentration of LH at the LF/A compared to the HBC (+) group.

**Table 3:** Subgroup Patient Characteristics and Clinical Laboratory Values

Variable	HBC (+)	HBC (-)	p-value
	(n = 9)	(n = 9)	
**Demographics**			
Age (y)	25 ± 7	23 ± 3	0.308
Time Since Diagnosis (y)	15 ± 10	13 ± 6	0.658
Height (cm)	166.3 ± 7.5	163.9 ± 6.9	0.484
Weight (kg)	80.2 ± 18.7	67.9 ± 18.3	0.177
BMI (kg/m2)	29.0 ± 6.4	25.2 ± 5.9	0.214
MAP (mm Hg)	91 ± 7	90 ± 6	0.949
**Clinical Laboratory Values**			
CRP (mg/L)	4.8 ± 4.3	8.0 ± 11.7	0.466
TC (mg/dL)	192 ± 42	179 ± 29	0.460
HDL (mg/dL)	70 ± 22	63 ± 18	0.444
LDL (mg/dL)	95 ± 33	97 ± 32	0.867
TRIG (mg/dL)	103 ± 67	67 ± 23	0.152
TC/HDL ratio	3.0 ± 1.2	3.0 ± 0.9	0.947
HbA1c (%)	8.3 ± 2.1	8.9 ± 2.1	0.509
FBG (mg/dL) – M/P	142 ± 60	132 ± 67	0.731
FBG (mg/dL) – LF/A	158 ± 53	158 ± 70	0.988
Insulin (uIU/mL) – M/P	1.3 ± 1.5	7.0 ± 18.3	0.386
Insulin (uIU/mL) – LF/A	1.4 ± 1.7	5.5 ± 14.5	0.419
Estradiol (pg/mL) – M/P	17 ± 14 (5–45)	42 ± 31 (12–114)	**0.041**
Estradiol (pg/mL) – LF/A	21 ± 35 (5-112)	108 ± 64* (28–210)	**0.002**
LH (mIU/mL) – M/P	3 ± 3 (1–11)	5 ± 3 (1–9)	0.203
LH (mIU/mL) – LF/A	5 ± 5 (0–12)	14 ± 9* (3–34)	**0.017**
Progesterone (ng/mL) – M/P	0.2 ± 0.1 (0.1–0.4)	0.5 ± 0.4 (0.1–1.3)	0.149
Progesterone (ng/mL) – LF/A	0.2 ± 0.1 (0.1–0.3)	1.1 ± 2.2 (0.1-7.0)	0.262

Data are presented as mean ± SD (Range); independent samples t test. Bold values indicate statistically significant differences between groups. *Significant difference from Menses within group. BMI = Body Mass Index, MAP = Mean Arterial Pressure, CRP = C-Reactive Protein, TC = Total Cholesterol, HDL = High-Density Lipoprotein, LDL = Low-Density Lipoprotein, TRIG = Triglycerides, HbA1c = Hemoglobin A1c, FBG = Fasting Blood Glucose, LH = Luteinizing Hormone

Figure 3B illustrates the FMD response across the menstrual cycle/time in both HBC (+) and HBC (-). There was a significant (*p* = 0.016; Cohen’s *d* = 0.683) group main effect. FMD was overall higher in HBC (+) compared to HBC (-), independent of menstrual cycle phase/time. In addition, a significant menstrual cycle phase main effect (*p* = 0.010; Cohen’s *d* = 0.632) was observed for FMD, which indicated that FMD was significantly reduced from M/P to LF/A, independent of birth control status. The parameters of the FMD test in HBC (+) and HBC (-) are presented in Table 4. There were no differences (all *p* > 0.05) in baseline diameter, peak diameter, and shear rate between groups. FMD/Shear was greater (*p* = 0.028; Cohen’s *d* = 0.582) in HBC (+) compared to HBC (-), independent of menstrual cycle phase/time. In addition, FMD/Shear was significantly lower (*p* = 0.003; Cohen’s *d =* 0.829) during the LF/A phase compared to M/P, independent of birth control status. Figure 3C illustrates the vasodilatory response to NTG across the menstrual cycle in HBC (+) and HBC (-). No differences in endothelial-independent dilation were observed (all *p* > 0.05) between groups or over time.

**Table 4:** Parameters of the FMD test throughout the menstrual cycle in patients with Type 1 Diabetes HBC (+) and HBC (-)

Variable	M/P			LF/A		
	HBC (+)	HBC (-)	p-value	HBC (+)	HBC (-)	p-value
Baseline diameter (cm)	0.281 ± 0.032	0.306 ± 0.027	0.091	0.285 ± 0.029	0.307 ± 0.027	0.122
Peak diameter (cm)	0.316 ± 0.035	0.334 ± 0.032	0.269	0.315 ± 0.032	0.325 ± 0.028	0.471
Shear (s^− 1^ AUC)	51,487 ± 20,264	52,242 ± 22,526	0.941	59,852 ± 18,080	45,892 ± 18,158	0.122
FMD/Shear (%/s^− 1^ AUC)	0.262 ± 0.087	0.186 ± 0.057	**0.045**	0.186 ± 0.084	0.131 ± 0.057	0.125

Values are mean ± SD. AUC = Area under the curve, FMD = Flow-mediated dilation. Bold values indicate between group significance at each menstrual cycle phase

### Effects of HBC on circulating biomarkers of oxidative stress and NO concentration in type 1 diabetes

The increase in NOx across the menstrual cycle phase/time was similar (*p* = 0.514) between groups. Despite this, the change in NO concentration appears to be two-fold greater (*p* = 0.817) in the HBC (-) group (ΔNOx = 0.995 ± 5.704 µM) compared to the HBC (+) group (ΔNOx = 0.453 ± 3.889 µM). Additionally, there was a significant time main effect (*p* = 0.030; Cohen’s *d* = 0.563) for TBARS, such that a two-fold increase in TBARS was observed in the HBC (-) group (ΔTBARS = 6.7 ± 11.5 µM) compared to the HBC (+) group (ΔTBARS = 3.8 ± 11.5 µM).

## Discussion

The cardiovascular protection observed in healthy premenopausal women has been linked, in part, to the vascular protective properties of circulating concentrations of estrogens [5]. Despite this beneficial role of estrogens, animal models have demonstrated that estradiol can promote vasoconstriction in the presence of diabetes [20]. Present findings in healthy, premenopausal women are consistent with previous reports that demonstrate the increase in estradiol across the menstrual cycle is accompanied by an improvement in vascular endothelial function. However, this investigation is the first to report, in humans, that endogenous production of estradiol contributes to lower endothelial function during the late follicular phase of the menstrual cycle in premenopausal women with type 1 diabetes, which is the opposite response observed throughout the menstrual cycle in healthy controls. Perhaps most important, the increase in estrogen appears to coincide with increases in TBARS, rather than a decrease in circulating concentrations of NO, which provides mechanistic insight into the impaired FMD throughout the menstrual cycle in type 1 diabetes. Further, FMD appears to be higher in patients with type 1 diabetes who use HBC compared to women with type 1 diabetes who do not use HBC. Taken together, these findings suggest that endogenous production of estradiol throughout the menstrual cycle has a negative impact on vascular endothelial function in women with type 1 diabetes, and the use of HBC may help in mitigating the estrogen-mediated vascular dysfunction in this patient population.

### The role of estradiol and oxidative stress on vascular endothelial function in type 1 diabetes

In healthy women, an increase in estradiol can help reduce oxidative stress and increase NO production, which can increase NO bioavailability and facilitate vasodilation [10, 11]. In addition, estradiol also has immuno-enhancing properties that can help promote angiogenesis [27, 28]. Further, estradiol exerts additional benefits on cellular health by not only stimulating the expression and activity of a wide array of antioxidant enzymes [12], but also reducing pro-inflammatory pathways through increases in anti-inflammatory processes [29]. Taken together, the overall improvement of endothelial function and vascular health that has been observed across the menstrual cycle in healthy, premenopausal women likely contributes to the substantial decrease in CVD risk. The data from the present investigation are consistent with previous reports that have documented that improvements in vascular function coincide with increasing concentrations of estradiol throughout the menstrual cycle in healthy women. However, this study demonstrates, for the first time, that the estrogen-mediated improvement in vascular endothelial function is lost in the presence of type 1 diabetes, a finding that persists after controlling for baseline differences in systemic inflammation (i.e., CRP) and BMI between groups. Importantly, no differences in fasting blood glucose or insulin were observed between menses and the late follicular phase of the menstrual cycle in patients with diabetes. Accordingly, these data support that acute glycemic management and glycemic control are not confounding the vascular and oxidative stress results. Nonetheless, future studies are needed to specifically determine if estrogens play a role on glycemic control and overall vascular health in type 1 diabetes.

Experimental data using animal models of type 1 diabetes indicate that estradiol may also signal an increase in oxidative stress and subsequent decrease in NO bioavailability [20]. Indeed, data from the present investigation demonstrate an increase in oxidative stress across the menstrual cycle only in women with type 1 diabetes, albeit a similar increase in circulating NO (Fig. 2). Although determining the mechanism for why estrogen increases oxidative stress in the presence of diabetes is beyond the scope of this initial clinical study, free radicals have a high affinity for NO and can reduce NO bioavailability. Accordingly, the increase in NO that was observed throughout the menstrual cycle, coupled with the estrogen-mediated increase in oxidative stress during the late follicular phase of the menstrual cycle in patients with type 1 diabetes, results in a decrease in NO bioavailability, ultimately causing endothelial dysfunction and an increase in CVD risk. Given that the effect sizes for the between group comparisons are robust, and the fact that every 1% reduction in FMD coincides with a 9% increase in future risk of CVD [30], these data suggest that premenopausal women with type 1 diabetes have a transient increase in CVD risk of 18% during the late follicular phase of the menstrual cycle compared to controls. Although the endothelial dysfunction may be transient, repeated impairment in vascular function every menstrual cycle could explain the overall higher CVD risk observed in women with type 1 diabetes compared to men with type 1 diabetes. Importantly, the vasodilatory response to sublingual nitroglycerin was similar between groups (Fig. 1C), which suggests that endothelial-independent vasodilation, or vascular smooth muscle cell function, is preserved in type 1 diabetes, and the estrogen-mediated vascular dysfunction is indeed endothelial-cell dependent. Taken together, therapeutic approaches to mitigate the estrogen-mediated increase in oxidative stress in type 1 diabetes are warranted to increase NO bioavailability and decrease overall CVD risk in response to endogenous production of estradiol across the menstrual cycle.

### Effects of HBC on vascular endothelial function and oxidative stress in women with type 1 diabetes

The use of HBC is widely used, and their impact on CVD risk has been a topic of debate. Although there are data to demonstrate that HBC does not cause any apparent increase in CVD risk [31], contrasting data in healthy women indicate that the use of HBC may have negative cardiovascular consequences [24]. In support of the latter, a 2.5 relative increased risk in CVD has been reported in apparently healthy users of HBC [32]. The present study utilized HBC to provide proof-of-concept of the effects of suppressing endogenous production of estradiol on endothelial function in type 1 diabetes. In fact, use of HBC can also suppress estrogen receptor activation and contribute to a reduction in NO-mediated vasodilation via the non-genomic pathway [33], which has the opposite response to the estrogen-mediated vasodilation and improvement in endothelial function typically observed during a normal menstrual cycle [14–18]. In support, concentrations of estradiol observed in the present study were overall lower at M/P in the HBC (+) group compared to the HBC (-) group. This finding is consistent with the concept that the use of HBC does not allow for a complete rebound of endogenous production of estradiol from its suppression during the active pill phase [34]. Despite the previously reported adverse data in healthy women, findings from the present investigation provide the first evidence to demonstrate that HBC may confer a benefit to vascular endothelial function in women with type 1 diabetes.

In the current study, FMD was overall higher in women with type 1 diabetes using HBC compared to those not using HBC (Fig. 3B), albeit both groups exhibiting a reduction in FMD from visit 1 (M/Placebo Pills) to visit 2 (LF/Active Pills). In fact, the higher FMD observed in the HBC (+) group was similar to the value observed in healthy controls (*p* = 0.105, data not shown) suggesting that HBC can potentially preserve endothelial function in women with type 1 diabetes. Importantly, no differences in fasting blood glucose or insulin were observed between patients who use and do not use HBC. Indeed, oxidative stress appears to increase over time in both the HBC (+) and the HBC (-) groups; however, the percent increase in TBARS was more than double that observed in the HBC (-) group, providing further support to an estrogen-mediated increase in oxidative stress in the presence of diabetes. In addition, a two-fold greater increase in NO concentration was also observed in the HBC (-) group, compared to the HBC (+) group. Although the greater increase in NO observed in the HBC (-) group may be related to the increase in endogenous production of estradiol, the estradiol-related increases in oxidative stress observed in this group likely plays a key role in reducing NO bioavailability and could provide insight into the lower FMD observed in the HBC (-) compared to the HBC (+) group. Nonetheless, given the increase in oxidative stress in both groups, future studies are certainly needed to determine alternative mechanisms that contribute to improvements in vascular health in patients with type 1 diabetes using HBC. Although the current study was unable to determine the exact mechanisms by which HBC protects vascular endothelial function in type 1 diabetes, the present data provide proof-of-concept that endogenous production of estrogen may play a negative role. It is important to note that both groups exhibited a similar progesterone profile; however, the HBC (+) group did not have an increase in LH during the LF/A visit as expected. Additionally, participants were taking a variety of oral HBC, and the results are inclusive of all four generations together. Therefore, future studies should investigate the differences between each generation of HBC and how they individually affect endothelial function. Although the LH surge can promote vasodilation in the ovarian vasculature [35], studies are certainly warranted to determine the role of systemic concentrations of LH on vascular health in type 1 diabetes. Further, there are data to support that the change in FMD throughout the menstrual cycle may not be due to the specific concentration during that phase. Rather, the vascular protection from endogenous production of estradiol may be due to the overall chronic cycling effect of estrogens [14] throughout multiple consecutive menstrual cycles. Although use of HBC prevents this cyclic pattern of sex-steroid hormone production, investigations that provide mechanistic insight into the higher FMD observed in patients with type 1 diabetes who use HBC are certainly warranted. Nonetheless, findings from the present investigation support the idea that suppressing circulating concentrations of endogenous estradiol may offer a cardiovascular benefit in women with type 1 diabetes; however, future randomized clinical trials are certainly warranted.

### Experimental considerations

It is important to acknowledge the relatively small sample size used for the main and sub-group analysis in the present study. Equally important; however, are the robust effect sizes that are presented for the primary outcomes in both analyses, which should reduce the potential concern of a small sample size. Nonetheless, given that this is the first study in humans to provide insight into the negative role of estradiol on endothelial function in the presence of diabetes, larger studies are certainly needed to expand and confirm the reported findings. In addition, the present investigation cannot determine cause and effect, and the precise mechanistic impact of estrogens on vascular health in type 1 diabetes remains elusive. Importantly, FMD was the primary outcome of the present investigation, and the present investigation was not powered to detect differences in circulating biomarkers of oxidative stress and/or concentrations of NO. In fact, we acknowledge that circulating biomarkers may be difficult to interpret in some cases and may not always reflect what is happening at the cellular level [14]. Nonetheless, the present findings do provide some mechanistic insight for the negative role of estrogen on oxidative stress in the presence of diabetes. Future bench and back-to-bedside mechanistic studies are certainly needed to elucidate how estrogens increase oxidative stress and contribute to impaired vascular health in the presence of diabetes. Moreover, the present study was conducted in young adult women with type 1 diabetes whose diabetes was relatively well-controlled. Accordingly, whether increasing age and advancing disease severity impacts estrogens effects on vascular health could not be answered with the present study and warrants further investigation.

## Conclusion

In conclusion, present findings are consistent with previous reports that indicate that there is an increase in FMD across the menstrual cycle in healthy, premenopausal women. However, for the first time in humans, the present investigation demonstrates that women with type 1 diabetes have an opposite response. Specifically, endogenous production of estradiol during the late follicular phase of the menstrual cycle contributes to an increase in oxidative stress that is accompanied by a reduction in FMD in women with type 1 diabetes. In addition, endothelial function appears to be higher in women with type 1 diabetes who use HBC compared to those women with type 1 diabetes who did not use HBC. These data support a negative role of estradiol on vascular function in the presence of diabetes. Future investigations providing treatment modalities that will reduce the estrogen-mediated increase in oxidative stress and improve vascular endothelial function in women with type 1 diabetes are certainly warranted.

## Data Availability

The datasets used during the current study are available from the corresponding author on reasonable request.

## Abbreviations

HBC: Hormonal birth control
HbA_1c_: Hemoglobin A_1c_
FMD: Flow-mediated dilation
CVD: Cardiovascular disease
M: Menses
LF: Late follicular
TBARS: Thiobarbituric acid reactive substances
NOx: Nitrate/nitrite
NO: Nitric oxide
CRP: C-reactive protein
BMI: Body mass index
SD: Standard deviation
SEM: Standard error of mean
MAP: Mean arterial pressure
TC: Total cholesterol
HDL: High-density lipoprotein
LDL: Low-density lipoprotein
TRIG: Triglycerides
FBG: Fasting blood glucose
LH: Luteinizing hormone
LIVEP: Laboratory of Integrative Vascular and Exercise Physiology
NTG: Nitroglycerin
AUC: Area under the curve
MDA-TBA: Malondialdehyde-thiobarbituric acid
M/P: Menses/placebo pill week
LF/A: Late follicular/active pill week

## References

[1] Roth GA, Mensah GA, Johnson CO, Addolorato G, Ammirati E, Baddour LM (2020). Global Burden of Cardiovascular Diseases and Risk factors, 1990–2019: Update from the GBD 2019 study. J Am Coll Cardiol.

[2] Mosca L, Barrett-Connor E, Wenger NK (2011). Sex/gender differences in cardiovascular disease prevention: what a difference a decade makes. Circulation.

[3] Lee SI, Patel M, Jones CM, Narendran P (2015). Cardiovascular disease and type 1 diabetes: prevalence, prediction and management in an ageing population. Ther Adv Chronic Dis.

[4] Lind M, Svensson AM, Kosiborod M, Gudbjörnsdottir S, Pivodic A, Wedel H (2014). Glycemic control and excess mortality in type 1 diabetes. N Engl J Med.

[5] Iorga A, Cunningham CM, Moazeni S, Ruffenach G, Umar S, Eghbali M (2017). The protective role of estrogen and estrogen receptors in cardiovascular disease and the controversial use of estrogen therapy. Biol Sex Differ.

[6] Huxley RR, Peters SA, Mishra GD, Woodward M (2015). Risk of all-cause mortality and vascular events in women versus men with type 1 diabetes: a systematic review and meta-analysis. Lancet Diabetes Endocrinol.

[7] de Ritter R, de Jong M, Vos RC, van der Kallen CJH, Sep SJS, Woodward M (2020). Sex differences in the risk of vascular disease associated with diabetes. Biol Sex Differ.

[8] Braffett BH, Bebu I, El Ghormli L, Cowie CC, Sivitz WI, Pop-Busui R (2022). Cardiometabolic risk factors and Incident Cardiovascular Disease events in women vs men with type 1 diabetes. JAMA Netw Open.

[9] Mihm M, Gangooly S, Muttukrishna S (2011). The normal menstrual cycle in women. Anim Reprod Sci.

[10] Khalil RA (2013). Estrogen, vascular estrogen receptor and hormone therapy in postmenopausal vascular disease. Biochem Pharmacol.

[11] White RE (2002). Estrogen and vascular function. Vascul Pharmacol.

[12] Borrás C, Gambini J, López-Grueso R, Pallardó FV, Viña J (2010). Direct antioxidant and protective effect of estradiol on isolated mitochondria. Biochim Biophys Acta.

[13] Bruno RM, Bianchini E, Faita F, Taddei S, Ghiadoni L (2014). Intima media thickness, pulse wave velocity, and flow mediated dilation. Cardiovasc Ultrasound.

[14] Harris RA, Tedjasaputra V, Zhao J, Richardson RS (2012). Premenopausal women exhibit an inherent protection of endothelial function following a high-fat meal. Reprod Sci.

[15] Adkisson EJ, Casey DP, Beck DT, Gurovich AN, Martin JS, Braith RW (2010). Central, peripheral and resistance arterial reactivity: fluctuates during the phases of the menstrual cycle. Exp Biol Med (Maywood).

[16] Williams MR, Westerman RA, Kingwell BA, Paige J, Blombery PA, Sudhir K (2001). Variations in endothelial function and arterial compliance during the menstrual cycle. J Clin Endocrinol Metab.

[17] Hashimoto M, Akishita M, Eto M, Ishikawa M, Kozaki K, Toba K (1995). Modulation of endothelium-dependent flow-mediated dilatation of the brachial artery by sex and menstrual cycle. Circulation.

[18] Brandão AHF, Serra PJ, Zanolla K, Cabral ACV, Geber S (2014). Variation of endothelial function during the menstrual cycle evaluated by flow-mediated dilatation of brachial artery. JBRA Assist Reprod.

[19] Shenouda N, Priest SE, Rizzuto VI, MacDonald MJ (2018). Brachial artery endothelial function is stable across a menstrual and oral contraceptive pill cycle but lower in premenopausal women than in age-matched men. Am J Physiol Heart Circ Physiol.

[20] White RE, Han G, Dimitropoulou C, Zhu S, Miyake K, Fulton D (2005). Estrogen-induced contraction of coronary arteries is mediated by superoxide generated in vascular smooth muscle. Am J Physiol Heart Circ Physiol.

[21] Britton LE, Alspaugh A, Greene MZ, McLemore MR (2020). CE: an evidence-based update on Contraception. Am J Nurs.

[22] Teal S, Edelman A (2021). Contraception Selection, Effectiveness, and adverse Effects: a review. JAMA.

[23] Rivera R, Yacobson I, Grimes D (1999). The mechanism of action of hormonal contraceptives and intrauterine contraceptive devices. Am J Obstet Gynecol.

[24] Kaminski P, Szpotanska-Sikorska M, Wielgos M (2013). Cardiovascular risk and the use of oral contraceptives. Neuro Endocrinol Lett.

[25] Harris RA, Nishiyama SK, Wray DW, Richardson RS (2010). Ultrasound assessment of flow-mediated dilation. Hypertension.

[26] Lakens D (2013). Calculating and reporting effect sizes to facilitate cumulative science: a practical primer for t-tests and ANOVAs. Front Psychol.

[27] Cutolo M, Capellino S, Sulli A, Serioli B, Secchi ME, Villaggio B (2006). Estrogens and autoimmune diseases. Ann N Y Acad Sci.

[28] Losordo DW, Isner JM (2001). Estrogen and angiogenesis: a review. Arterioscler Thromb Vasc Biol.

[29] García-Gómez E, Vázquez-Martínez ER, Reyes-Mayoral C, Cruz-Orozco OP, Camacho-Arroyo I, Cerbón M (2019). Regulation of inflammation pathways and Inflammasome by Sex Steroid Hormones in Endometriosis. Front Endocrinol (Lausanne).

[30] Green DJ, Jones H, Thijssen D, Cable NT, Atkinson G (2011). Flow-mediated dilation and cardiovascular event prediction: does nitric oxide matter?. Hypertension.

[31] Katerndahl DA, Realini JP, Cohen PA (1992). Oral contraceptive use and cardiovascular disease: is the relationship real or due to study bias?. J Fam Pract.

[32] Stampfer MJ, Willett WC, Colditz GA, Speizer FE, Hennekens CH (1988). A prospective study of past use of oral contraceptive agents and risk of cardiovascular diseases. N Engl J Med.

[33] Chen Z, Yuhanna IS, Galcheva-Gargova Z, Karas RH, Mendelsohn ME, Shaul PW (1999). Estrogen receptor alpha mediates the nongenomic activation of endothelial nitric oxide synthase by estrogen. J Clin Invest.

[34] Brenner PF, Mishell DR, Stanczyk FZ, Goebelsmann U (1977). Serum levels of d-norgestrel, luteinizing hormone, follicle-stimulating hormone, estradiol, and progesterone in women during and following ingestion of combination oral contraceptives containing dl-norgestrel. Am J Obstet Gynecol.

[35] Duffy DM, Ko C, Jo M, Brannstrom M, Curry TE (2019). Ovulation: parallels with inflammatory processes. Endocr Rev.

